# Job characteristics that enrich clinician-educators’ career: a theory-informed exploratory survey

**DOI:** 10.1080/10872981.2022.2158528

**Published:** 2022-12-22

**Authors:** M. Hossein Tcharmtchi, Shelley Kumar, Jennifer Rama, Brian Rissmiller, Danny Castro, Satid Thammasitboon

**Affiliations:** a Department of Pediatrics, Baylor College of Medicine, Houston, TX, USA; b Center for Research, Innovation and Scholarship in Health Professions Education (CRIS), Texas Children’s Hospital, Houston, TX, USA

**Keywords:** Job satisfaction, job motivation, wellness, professional development, human resources

## Abstract

Clinician educators (CEs) play a vital role in helping academic institutions achieve the missions of patient care, education and research. The driving forces that motivate pediatric CEs in professional growth and personal satisfaction remain unexplored. An exploratory survey research to investigate the job characteristics and factors that motivate CEs to pursue professional growth with personal satisfaction. Using the Job Characteristics Model (JCM) as a framework, we developed a 22-item survey comprised of the JCM derived Job Diagnostic Survey, Global Job Satisfaction scales and demographics. We collected data from January 2020 to March 2020 from self-identified pediatric CEs (with and without educational leadership roles) through a survey recruitment service. Given no data on total number of CEs in the survey pool, response rate was unknown. Job characteristics in the core job dimensions of meaningfulness, autonomy, and performance feedback, as well as, the derived Motivating Potential Score (MPS), were analyzed using descriptive statistics and regression models. From 201 respondents, including 55 education leaders, >70% were satisfied with patient care, teaching, and mentoring while <40% were satisfied with administrative and scholarly activities. Meaningfulness (in some areas), autonomy (patient care/teaching), and internal feedback (all areas) had significant effects on job satisfaction. In regression analysis, skill variety, feedback, and years of experience were associated with higher job satisfaction, and the MPS was a predictor of total job satisfaction. The JCM can be utilized to understand CE’s motivations and needs within their workplace and guide professional development via job enrichment efforts.

## Introduction

Clinician-educators (CEs) play a vital role in supporting the achievement of academic medicine’s tripartite mission: clinical service, education and research [1]. Most CE’s tasks and responsibilities have exponentially increased over recent years [2,3]. CEs are expected to pursue academic tasks and scholarly activities in the face of substantial clinical responsibilities [4,5]. CEs with leadership positions (e.g., program directors) are faced with increasing responsibilities posed by regulatory agencies and public demands [5–7]. Despite a large body of literature on well-being, burnout and job satisfaction among physicians, these issues remain unaddressed for this particular group.

Maslow’s need-hierarchy and Herzberg’s two-factor theories are commonly applied in the human resources literature as it relates to job satisfaction [8,9]. Maslow posits that employees have five levels of needs (physiological, safety, social, self-esteem, and self-actualizing), and that lower-level needs have to be satisfied before the next level of need could motivate them. Herzberg describes factors such as achievement, recognition, promotion and various intrinsic aspects induce motivation. Hygiene factors such as supervision, salary, work environment and relationships do not increase job satisfaction, but their absence causes dissatisfaction. From the medical education literature, a facet-specific job satisfaction instrument developed by Beasley has been studied in residency program directors in internal medicine, radiology and obstetrics and gynecology [10–12]. Facets including working with residents, colleague relationships, resources, patient care, pay, and promotion increased job satisfaction.

We posit that studying job satisfaction and motivation together, without clear demarcation adds more layers of complexity to the issue. *Job satisfaction* is an individual’s emotional response to current job condition whereas *motivation* is an internal force that accounts for the level and direction and persistence of effort expended at work, the driving force to pursue and satisfy individual needs for growth and wellness [13]. Robust literature has shown that the relation of job satisfaction, motivation and performance is more circular than a linear process due to interdependent relationship of the variables [14–16]. Two-Factor theory classifies hygiene and motivation as two separate factors, however, also highlights the complex relationships between motivation and satisfaction, and the sources of job satisfaction and dissatisfaction. Job satisfaction and employee motivation are inter-dependent, and both contribute to the success of an organization. Creating conditions to satisfy an employee’s needs may not help an individual achieve overall job satisfaction or performance. Either satisfactions and dissatisfactions about one’s job can strengthen employee motivation in different contexts. Therefore, gaining insights into the dynamic of these two variables may help us find ways to enrich professional development of CEs.

We assert that pediatric CEs represent a unique group of clinicians with strength to pursue personal and professional growth. If our overarching goal is to promote growth and wellness, we need to move beyond studying job (dis)satisfaction, and focus efforts on positive strength-based psychology approach that emphasizes what motivates CEs, what CEs really care about, and their positive core values, rather than on dissatisfaction avoidance and burn out [17–19]. Thus, we set the premise for this study to explore employee motivation and job satisfaction. We aimed to gain insights from pediatric CEs about factors that enabled sustained commitment to fulfilling their roles as integral parts of an academic institution as well as compare these factors between those with and without leadership roles. Through a theory-informed survey, we are poised to derive a strategic framework to enrich professional development of CEs.

## Materials and methods

### Setting and population

This was an exploratory, cross-sectional survey to investigate the job characteristics and factors that motivate CEs to pursue professional growth with personal satisfaction. We accessed a large pool of physicians in the United States through a third-party survey company to facilitate a nationwide recruitment of pediatric CEs from January 2020 to March 2020. We defined a CE as a clinician whose primary roles were in clinical practice, practice-related activities and education-related activities. The study was approved by the Baylor College of Medicine institutional review board

### Theoretical framework

**Figure 1.**
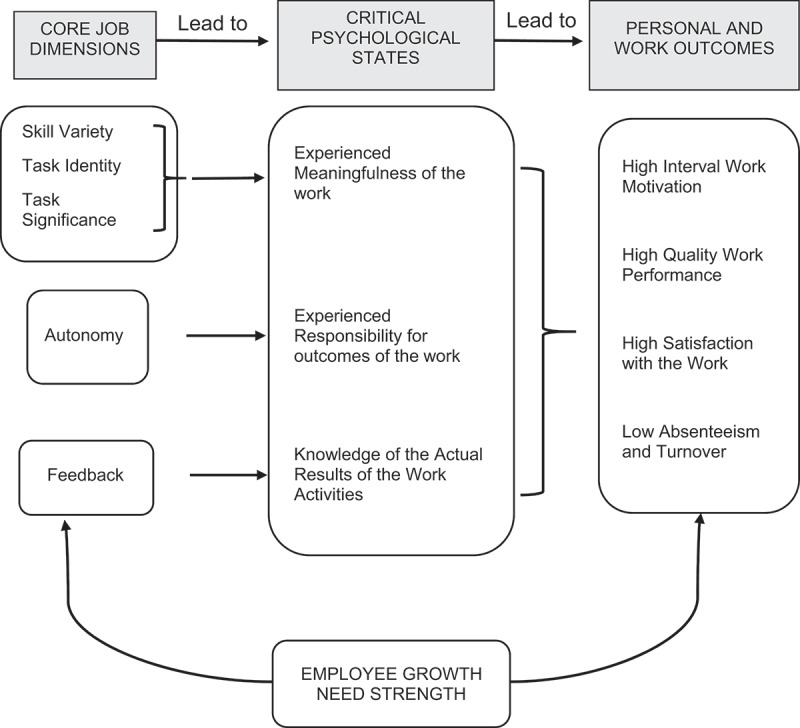
The Job characteristics model posits experiencing meaningfulness of the work, sense of responsibility for the outcome of the work and knowledge of results of the work activities as three critical psychological states needed for satisfactory personal and work outcomes. Five core job dimensions; skill variety, task identity task significance, autonomy and feedback can induce these three states. The figure has been reproduced from Hackman and Oldham with permission from American psychological association.

After exploration of the literature, we identified the Job Characteristic Model (JCM) as an appropriate theoretical framework for the study (Figure 1) [20]. The JCM posits that satisfactory personal and work outcomes can result only when an individual has experienced all ‘three *critical psychological states’*:1) meaningfulness of the work, 2) responsibility for the outcome of the work and 3) knowledge of results of the work activities. These psychological states correspond to five core job dimensions. Meaningfulness of work is enhanced by the presence of first three core job dimensions: *Skill Variety* – the degree that a job requires a variety of different activities involving the use of different skills and talents, *Task Identity* – requiring completion of a ‘whole and identifiable’ task, seeing through a task from the start to finish, and *Task Significance* – importance and impact of the task on the lives and well-being of others. Another dimension, *Autonomy*, induces realization of substantial freedom and discretion for the outcome of the work. The last dimension, *Feedback*, refers to *internal feedback* from the job itself and *external feedback* from others receiving direct and clear information about the effectiveness of the performance as well as the knowledge of the actual work outcome. Analyzing these dimensions can provide insights to what motivate CEs. The Job Diagnostics Survey (JDS) is a tool based on the JCM [20–24]. The JDS generates a ‘Motivating Potential Score’ (MPS) following the formula:

$$MPS=\frac{\left(Skill\;Variety+Task\;Identity+Task\;Significance\right)}{3}\;x\;Autonomy\;x\;Feedback$$

### Survey development

**Figure 2.**
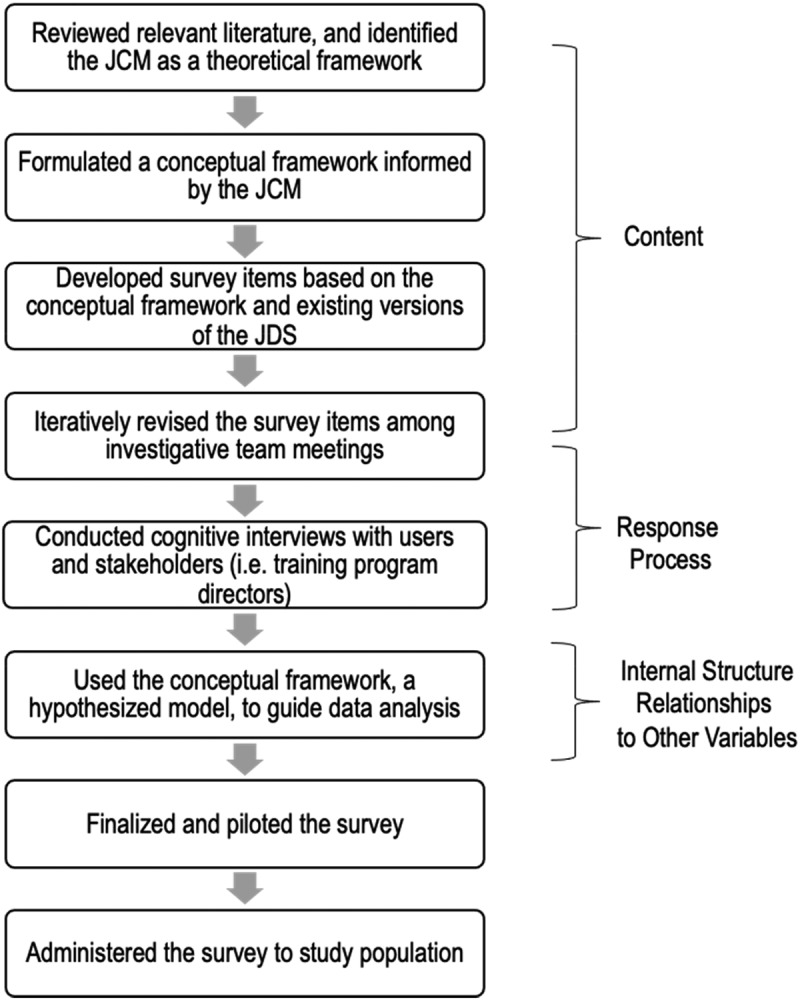
Study flow.

We employed a systematic approach to survey development [25], guided by Messick’s validity framework [26]. see Figure 2: Study Flow represents the conceptual framework supplement 1 used to guide the development of survey questions and hypothesized model for data analysis. The survey had three components.

We employed a systematic approach to survey development using the Job Characteristics Model as a theoretical framework. Through various steps of the survey development, we gathered and planned to ascertain validity evidence according to the Messick’s validity framework (i.e. Content, Response Process, Internal Structure and Relationship to Other Variables). JCM: Job Characteristics Model, JDS: Job diagnostic Survey

1. Demographics: This section elicited respondents’ characteristics.
2. Job Characteristics: The original JDS had been studied resulting in multiple versions for considerable number of contexts [27–31]. Three investigators integrated multiple JDS versions for this survey. We aimed to create survey items, guided by the JDS construct, and modified for an exploratory study of CEs, with and without leadership roles, rather than to create another JDS derivative. To simplify the survey, the behavioral anchors were shortened while preserving the original meanings. In line with the aforementioned positive psychology approach, all negative items were revised to positive statements.
3. Global Ratings on Job Satisfaction: This item gauged respondents’ general perceptions about various job-related tasks.

All investigators participated in survey iterations. To gather additional *content* validity, we collected feedback from 15 experts in education and program leaders. To ascertain *response process* validity, cognitive interviews were conducted with 12 CEs from different subspecialties with iterative modifications for terms, clarity and format. This resulted in the following changes: the job characteristics section was reduced to seven items (Table 1); one double barrel question related to Task Identity was revised to two items; the 10-item agreement scale questions (i.e., JDS Part Two) were discarded as all participants perceived them redundant; and the Likert scale was changed from 7 to 5 points based on suggested best practices [32].

**Table 1.** The Job characteristics items of the survey. The items are based on the job characteristics model comprising five job dimensions.

Core Job Dimension	Item
Skill Variety	To what extent does being a clinician-educator require you to use a variety of your skills and talents?
Task Identify	Within your role as a clinician-educator, to what extent do you see your work as being well-defined (i.e., with an obvious beginning to end)? Within your role as a clinician-educator, to what extent are the results of your work clearly visible and identifiable?
Task Significance	Overall, to what extent do the results of your work as a clinician-educator have a significant impact on well-being of other people? (trainees, patients, and families or other healthcare providers)
Autonomy	As a clinician-educator, to what extent do you have autonomy in performing your work?
Feedback	
External Indicator	Based on what success means to you as a clinician-educator, to what extent do you have external indicators (e.g., feedback and recognition from others) to accurately inform you about your performance?
Internal Indicator	Based on what success means to you as a clinician-educator, to what extent do you have internal indicators (e.g., feeling rewarded and accomplished) to accurately inform you about your performance?

### Survey administration

We piloted the survey and monitored the first 20 responses to ascertain integrity of the data and the data collection process. The time required to complete the survey was approximately 10 minutes on the SurveyMonkey Inc (San Mateo, California, USA). We used a survey data collection service (SurveyHealthCare) to facilitate distribution of the survey to their pool of 12,000 pediatricians. This service dispensed a gift card to each respondent as an incentive upon completion of the survey, and thus the respondents’ identifications were anonymous to the investigators. The data collection was concluded once the study target of 201 respondents resembling the population of our interest were reached. The service did not have information about the proportion of CEs in their survey pool, and thus the response rate was unknown.

### Data analysis

Data are described as frequency (%) and median (interquartile range, IQR) for continuous variables. Chi-square/Fisher exact tests were used to assess differences in count data. We compared the variables between the CEs with and without education leadership roles using Wilcoxon rank-sum tests. Both bivariate and multivariate models were constructed to investigate the associations between independent variables (i.e., demographics, job characteristics, job dimensions), MPS and total job satisfaction score (summation of ratings in all areas). We used the SAS (version 9.3, SAS Institute Inc., Cary, North Carolina) *Glmselect* procedure to build multivariate models of job satisfaction outcome. Only independent variables with p-value of 0.1 or less were retained in the model. All p-values were two sided, with the alpha level set at 0.05. Sample size was estimated using a rule of thumb [33,34], 10:1 ratio of participants to predictors (minimum required for regression equation using six or more predictors). Since no more than 15 parameters were tested simultaneously, a sample size of 201 (150 + 50 for possible incomplete responses) was deemed appropriate. This sample size also meets the recommended minimum 200 subject requirement for regression analysis. We set a priori to recruit 1:3 ratio of CEs with education leadership roles to those without.

## Results

### Descriptive statistics

Fifty-five from total of 201 respondents (27%) reported having an education leadership role (e.g., program director, faculty development, vice chair education). Only 29% reported having protected time for educational commitments; of those having protected time, 43% were education leaders. Almost half (47%) of those also reported having leadership positions outside education. Respondents rated all five job-related tasks positively from ‘some extent’ to ‘great extent’ (3–5 out of 5). The majority of respondents reported being satisfied with their job prospects in patient care (80%), teaching (77%) and mentoring (71%) tasks. Strikingly low proportions were satisfied with administrative (30%) and scholarly activity (40%) tasks. Education leaders, more than other CEs, reported having satisfaction in teaching (87% vs.73%, p = .04) and scholarly activity (54% vs. 35%, p = .02). Compared to other CEs, educational leaders reported having more protected time (44% vs. 23%, p = .002), and leadership roles outside of education (60% vs. 43%, p = .04). Respondents holding leadership roles outside of education were also more satisfied with their scholarly activity (49% vs. 32%, p = .02). Table 2 provides additional findings.

**Table 2.** Descriptive statistics of responses.

Variable		Clinician-Educators n (%)		p-value
	Overall (n=201)	Not Educational Leaders (n=146)	Education Leaders (n=55)	
Age				
25 to 34	13 (7)	12 (9)	1 (2)	0.06^a^
35 to 44	57 (29)	40 (28)	17 (32)	
45 to 55	53 (27)	41 (29)	12 (22)	
55 to 64	52 (27)	32 (23)	20 (37)	
65 to 74	19 (10)	17 (12)	2 (4)	
Do not want to disclose	2 (1)	0 (0)	2 (4)	
Missing	5	4	1	
Gender	85 (43)	67 (47)	18 (33)	0.14^b^
Female	109 (56)	75 (53)	34 (63)	
Male	2 (1)	0 (0)	2 (4)	
Do not want to disclose	5	4	1	
Missing				
Advanced Degree(s)				
No	134 (68)	102 (72)	32 (59)	0.22 ^a^
Pursuing	8 (4)	5 (4)	3 (6)	
Yes	54 (28)	35 (25)	19 (35)	
Missing	5	4	1	
Job Satisfaction				
Patient Care	156 (80)	112 (79)	44 (83)	0.52 ^b^
Teaching	151 (77)	104 (73)	47 (87)	0.04 ^b^
Mentoring	137 (71)	96 (69)	41 (77)	0.23 ^b^
Administrative Work	58 (30)	40 (29)	18 (33)	0.54 ^b^
Scholarly Activity	77 (40)	49 (35)	28 (54)	0.02 ^b^
Other Leadership Role(s)				
Yes	95 (47)	62 (43)	33 (60)	0.04 ^b^
Protected time				
Yes	56 (29)	32 (23)	24 (44)	0.002 ^b^
Variable	Median (Interquartile Range)			p-value
Core Job Dimension				
Skill Variety	4 (4–5)	4 (4–5)	5 (4–5)	0.052^c^
Task Identity-				
Well defined work	3 (3–4)	3 (3–4)	4 (3–4)	0.056 ^c^
Visible and identifiable	4 (3–4)	3.5 (3–4)	4 (3–4)	0.28 ^c^
Task Significance	4 (3–4)	4.5 (3–4)	4 (3–4)	0.46 ^c^
Autonomy	4 (4–5)	4 (4–5)	4 (4–5)	0.108 ^c^
Feedback				
Internal Indicator	4 (3–4)	4 (3–4)	4 (3–4)	0.946 ^c^
External Indicator	3 (3–4)	3 (3–4)	4 (3–4)	0.005 ^c^
Motivating Potential Score	58 (44–77)	58 (44–75)	59 (44–82)	0.45 ^c^
Number of years of experience	12 (5–20)	13 (5–20)	12 (5–20)	0.983 ^c^

All the p-values compare ‘Not Education leaders’ group to ‘Education leader’ group.

P-values calculated using a Fisher-exact test, b Chi square test, cWilcoxon rank sum test.

Job Satisfaction represents ‘Very satisfied (4)’ and ‘Extremely (5) satisfied’ in each area.

Some percentages do not add up to 100% due to rounding.

### Motivating Potential Score (MPS) and core job dimensions

Overall, the median MPS was 58 (IQR 44–77, maximum score 125). There was no difference comparing the MPS between the CEs with those without education leadership roles. The median MPS was higher among those who were satisfied in each of the five areas than those who were not. Analyzing job dimensions, all components of meaningfulness of job had significant effects on job satisfaction as followings: *Skill Variety* on teaching, mentoring and scholarly activities; *Task Identity (visible and identifiable)* on patient care, teaching, mentoring, and administrative work, while *Task Identity (well defined)* had significant effect only on teaching; and *Task Significance* on patient care and teaching. *Autonomy* had significant effect on patient care and teaching. *Internal Indicator of Feedback* had significant effect on all areas whereas *External Indicator of Feedback* had significant effect on all areas except patient care (Table 3).

**Table 3.** Core job dimensions and job satisfaction (median with interquartile range).

Variable Median (IQR)	Job Satisfaction^a^														
	Patient Care			Teaching			Mentoring			Administrative Work			Scholarly Activity		
	Yes	No	p	Yes	No	p	Yes	No	p	Yes	No	p	Yes	No	p
Motivating Potential Score	63 (48–80)	40 (30–59)	*	64 (48–82)	44 (30–58)	*	64 (48–83)	44 (37–65)	*	64 (50–84)	56 (40–75)	*	64 (50–84)	55 (40–73)	*
Skill variety	4 (4–5)	4 (4–5)		4 (4–5)	4 (3–5)	*	4 (4–5)	4 (3.5–5)	*	4 (4–5)	4 (4–5)		5 (4–5)	4 (4–5)	*
Task Identity- Well Defined	4 (3–4)	3 (3–4)		4 (3–4)	3 (2–4)	*	4 (3–4)	3 (3–4)		4 (3–4)	3 (3–4)		4 (3–4)	3 (3–4)	
Task Identity-Visible/Identifiable	4 (3–4)	3 (2–4)	*	4 (3–4)	3 (2–4)	*	4 (3–4)	3 (2.5–4)	*	4 (3–4)	3 (3–4)	*	4 (3–4)	3 (3–4)	
Task Significance	4 (4–5)	4 (3–5)	*	4 (4–5)	4 (3–5)	*	4 (4–5)	4 (3–5)		4 (4–5)	4 (4–5)		4 (4–5)	4 (4–5)	
Autonomy	4 (4–5)	4 (3–5)	*	4 (4–5)	4 (3–5)	*	4 (4–5)	4 (4–5)		4 (4–5)	4 (4–5)		4 (4–5)	4 (4–5)	
Feedback-Internal	4 (3–4)	3 (3–4)	*	4 (3–5)	3 (3–4)	*	4 (4–5)	3 (3–4)	*	4 (3–5)	4 (3–4)	*	4 (4–5)	4 (3–4)	*
Feedback-External	3 (3–4)	3 (2–4)		3 (3–4)	3 (2–3)	*	3 (3–4)	3 (2–3.5)	*	4 (3–4)	3 (3–4)	*	4 (3–4)	3 (3–4)	*

^a^Job satisfaction with patient care, teaching, mentoring, administrative work, scholarly activities was dichotomized as not satisfied (very dissatisfied, moderately dissatisfied, neither dissatisfied nor satisfied) and satisfied (moderately satisfied, very satisfied).

*P-values significant using Wilcoxon rank sum test.

### Regression analyses

In bivariate analyses, age had significant effect on MPS whereas leadership roles, having protected time had no effect on total job satisfaction score or MPS. Number of years of experience had a small but significant correlation with total job satisfaction score and MPS (Supplement 2). In the multivariate regression model 1, using the job dimensions and other descriptive predictors of total job satisfaction, we found skill variety, task identity (well-defined), number of years as clinician-educator, and internal feedback having significant effect (R^2^ = 0.275). In model 2, the MPS along with age and holding leadership position outside of education had significant effect on total job satisfaction (R^2^ = 0.198) (Supplement 3).

## Discussion

Defining job characteristics that motivate CEs is a critical step to fostering their professional development and could, in turn, impact missions of the institutions. This exploratory survey yielded findings consistent with the JCM focusing on job enrichment through features of the job that motivate CEs to pursue professional growth with personal satisfaction. Our finding showed the Motivating Potential Score, calculated from the JCM core job dimensions, was associated with total job satisfaction, with all job-related tasks including patient care, teaching, mentoring, administrative work and scholarly activity. The individual core job dimensions had noteworthy effects, to varying extent, on reported job satisfaction and in various job-related tasks. Administrative and scholarly activities were the two least satisfying areas by a large portion of CEs in this study. Holding leadership responsibilities, either in education or other areas, was associated with job satisfaction, particularly in teaching and scholarly activities.

This study represents the first application of the JCM to CEs, though substantial literature exists in profit and non-profit organizations like teachers, social workers and healthcare professionals [35–37]. In this current study, the JCM was purposefully used as an alternative theory-informed framework, a complementary ‘lens’, to existing approaches to promoting wellness and addressing physician burnout. Maslach’s burnout inventory-human services survey has been used extensively to define burnout [38]. When individuals are ‘burned out’, they may feel overwhelmed, emotionally drained, physically exhausted, become cynical related to their jobs, unable to recognize meaning and identity in their work, and fail to acknowledge personal achievements. According to JCM, learning (skill variety), meaning (task significance) and identity (task identity) are affordances for job motivation and critical to enriching the work environment. Robust continuing professional development activities can engage CEs, either as individuals or communities of educators, to learn, share and apply new skills to navigate their work tasks successfully. Recognizing or rewarding educators’ achievements, at various public venues, help highlight the significance and the identity of being a CE. Autonomy and feedback, fostered by the organization or community, facilitate its members toward realizing or enabling job characteristics that enrich their work. Having community forums or peer support programs for educators with various interests can open great opportunities for engagement in teaching and scholarly activities, as well as exchanges of ideas and feedback. Assessing how CEs realize their job characteristics using this survey allows for a ‘diagnostic’ purpose to identify specific job dimensions requiring interventions from the individual and organizational perspectives.

We were interested in gaining viewpoints of CEs with education leadership roles given the challenges created by increasing demands fulfilling their leadership roles [39–41]. Our findings revealed that serving in a leadership position was not predictive of higher ‘total’ job satisfaction. Unlike other CEs in this study, however, these education leaders were very satisfied with their teaching and scholarly activities, the tasks that foster learning, meaning and identity as a clinical teacher in academia [42,43]. These findings are consistent with literature on job satisfaction among physicians [10–12]. Relative to other CEs, more education leaders reported having protected time, held other leadership roles, as well as, a high perception of external indicators of feedback. These differences may explain reasons they were recognized for their contributions in teaching and scholarly activities.

Local institutions or professional associations can use this study to realize what CEs perceive as common values and their associated needs for growth within the CE role. Using the JCM to guide the needs assessment within the local context can result in strategies to enrich the work environment and motivate CEs. For instance, areas for improvement may include addressing administrative work, supporting scholarly activities, and fostering skills development. Institutional efforts should be directed toward creating an environment where CEs are provided professional growth opportunities motivating them to meaningfully engage with trainees. Furthermore, formal recognition of CE’s efforts whether as financial incentives, protected time, promotion, and/or award systems are essential to motivating and sustaining CEs workforce.

This study has some limitations. Despite a rigorous approach to survey development, interpretation of survey items depends on individuals. Social desirability bias might have influenced their responses. We targeted CEs in pediatrics specialties and therefore our results have limited generalizability. The respondents may represent individuals who had free time or particular interest in the subject matter than other CEs. Our population represents busy clinicians, thus we opted to eliminate many items related to demographics and other individual characteristics to keep the survey concise. We do not have a true survey response rate. As an exploratory study of the JCM, our sample was set a priori for statistical analyses and recruited from a large pool of pediatricians to attain appropriate spectrum of CEs. We have not yet gathered empirical evidence to substantiate the normative value of the MPS given its first use in this context. The CEs in our study appeared to be quite satisfied with their job-related tasks even with the inter-quartile range of 44–77 on the MPS.

We gathered validity evidence to support the results of this survey through the development, setting the foundation for future application of the JCM in the CE’s context. As we employed a well-established theory into the survey, we hope our study can support a transferable approach to studying job motivation and satisfaction, and the findings provide theoretically grounded practices to enrich professional development of CEs. More studies are needed to substantiate the practical use of the MPS and contributions of interventions targeting different core job dimensions.

## Conclusion

This study is the first application of the JCM framework to CEs, and our results substantiate the hypothesized model, or associations, of JCM core job dimensions and job satisfaction. The JCM framework can be used to determine needs within the workplace, and guide professional development via job enrichment efforts

## Supplementary Material

Supplemental Material
