# Supplementary material for: Job characteristics that enrich clinician-educators’ career: a theory-informed exploratory survey
Source: Med Educ Online. 2022 Dec 22;28(1):2158528. doi: 10.1080/10872981.2022.2158528 (PMC9793935; doi:10.1080/10872981.2022.2158528)
Supplement: Supplemental Material [file ZMEO_A_2158528_SM2569.zip › Supplementary files/New Supplement 1.docx]

| Descriptive Predictor  *(Antecedents: Independent variables)* | Core Job Dimension  (Conditions: *Independent variables)* |  | Critical Psychological State  *(Processes*  *based on the JCM)* |  | Satisfaction  (Outcomes: Dependent variables) |
| --- | --- | --- | --- | --- | --- |
| Demographics  Years of Experience  Leadership Role(s)  Protected Time | Skill Variety |  | Meaningfulness of the Work |  | Patient Care |
|  | Task Identity (well-defined/ visibility) |  |  |  | Teaching |
|  | Tasks Significance |  |  |  | Mentoring |
|  | Autonomy |  | Responsibilities  for Outcomes |  | Administrative Work |
|  | Feedback (Internal/ External Indicator) |  | Knowledge of Outcomes |  | Scholarly Activities |

Supplement table 1. The Conceptual Framework for the Survey.

This proposed conceptual framework, based on the Job Characteristics Model, was formulated and used to guide the development of the survey items, and served as a hypothesized model for the data analyses. The arrows represent the hypothesized associations between variables.
