# Supplementary material for: Job characteristics that enrich clinician-educators’ career: a theory-informed exploratory survey
Source: Med Educ Online. 2022 Dec 22;28(1):2158528. doi: 10.1080/10872981.2022.2158528 (PMC9793935; doi:10.1080/10872981.2022.2158528)
Supplement: Supplemental Material [file ZMEO_A_2158528_SM2569.zip › Supplementary files/Supplement Survey.docx]

Job Diagnostic Survey for Clinician-Educators

1. Do you have a formal leadership role(s) in education (program director, associate program director, clerkship director, etc.)?

Yes

No


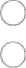


If yes, please specify your leadership role(s).

1. Do you have a leadership role(s) in other areas (medical director, quality improvement, patient safety officer, etc.)?

Yes

No


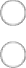


If yes, please specify your leadership role(s).

1

Job Diagnostic Survey for Clinician-Educators

Part A: Job Characteristics

**Please describe your job satisfaction pertaining to your role as a Clinician-Educator (CE). *If you have a formal leadership role in education please use that role as your primary point of reference to answer the following questions:***

1. As a CE, to what extent do you have autonomy in performing your work?

1- Not at all 2- To a small extent 3 - To some extent 4- To a moderate extent 5 - To a great extent


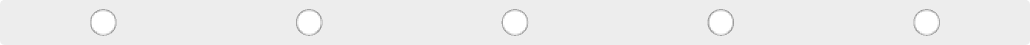


1. Within your role as a CE, to what extent do you see your work as being well defined (i.e. with an obvious beginning and end)?

1- Not at all 2- To a small extent 3- To some extent 4- To a moderate extent 5- To a great extent


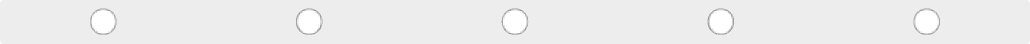


1. Within your role as a CE, to what extent are the results of your work clearly visible and identifiable?

1- Not at all 2- To a small extent 3- To some extent 4- To a moderate extent 5- To a great extent


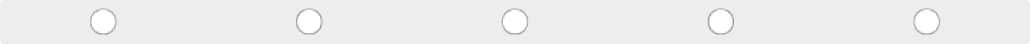


1. To what extent does being a CE require you to use a variety of your skills and talents?

1- Not at all 2- To a small extent 3- To some extent 4- To a moderate extent 5- To a great extent


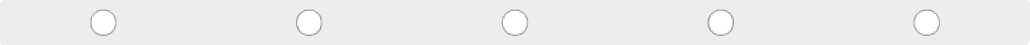


1. Overall, to what extent do the results of your work as a CE have a significant impact on the well being of other people? (trainees, patients,and families or other healthcare providers)

1 - Not at all 2 - To a small extent 3 - To some extent 4 - To a moderate extent 5 - To a great extent


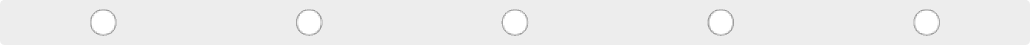


2

1. Based on what success means to you as a CE, to what extent do you have external indicators (e.g. feedback and recognition from others) to accurately inform you about your performance?

1 - Not at all 2 - To a small extent 3 - To some extent 4 - To a moderate extent 5 - To a great extent


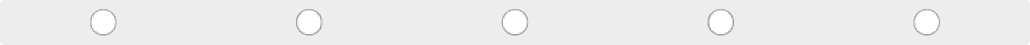


1. Based on what success means to you as a CE, to what extent do you have internal indicators (e.g. feeling rewarded and accomplished) to accurately inform you about your performance?

1 - Not at all 2 - To a small extent 3 - To some extent 4 - To a moderate extent 5 - To a great extent


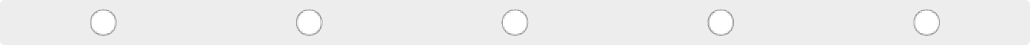


**Part B: Reflective comments**

1. Describe what you value most about yourself, your work and your institution.
2. Describe how do you see yourself and your institution grow in the future.
3. Think back through your career as a CE, locate a high point, when you felt most effective, engaged or successful. Describe how you felt and what made the situation possible.

3

Job Diagnostic Survey for Clinician-Educators

**Part C**

**Section I: Please provide following information about yourself.**

* 13. Please describe approximately the composition of your total time spent on the following (Should add up to 100%):

Patient Care

Teaching/Mentoring

Educational Administrative Work

Scholarly Activities: (research, curriculum development, advocacy, scholarly writing, etc.)

Other Administrative Work

1. Do you have a formal “protected ” time to serve in educational activities?

Yes No


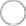


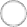


If yes, how much (in FTE)? (e.g., 0.2, 0.4 ).

1. Do you have an additional certificate(s) or advanced degree(s) in addition to your medical degree?

| 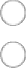 | Yes  No | 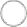 | Pursuing |
| --- | --- | --- | --- |

If yes, please specify:

**Section II. The following questions pertain to your job satisfaction as a CE.**

4

1. Please rate your satisfaction with your job prospects (i.e. the outlook for career advancement and/or success) at the current institution.

3 - Neither

2 - Moderately dissatisfied nor 4 - Moderately

1 - Very dissatisfied dissatisfied satisfied satisfied 5 - Very satisfied

|  |  |  |  |  |  |  |  |  |  |  |
| --- | --- | --- | --- | --- | --- | --- | --- | --- | --- | --- |
| Patient Care | 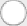 |  | 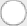 |  | 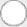 |  | 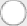 |  | 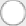 |  |
|  |  |  |  |  |  |  |  |  |  |  |

| Teaching | 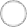 | 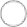 | 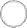 | 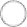 | 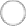 |
| --- | --- | --- | --- | --- | --- |

|  |  |  |  |  |  |  |  |  |  |  |
| --- | --- | --- | --- | --- | --- | --- | --- | --- | --- | --- |
| Mentoring | 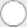 |  | 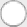 |  | 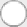 |  | 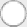 |  | 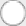 |  |
|  |  |  |  |  |  |  |  |  |  |  |

| Administrative work | 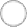 | 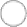 | 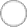 | 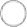 | 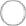 |
| --- | --- | --- | --- | --- | --- |

|  |  |  |  |  |  |  |  |  |  |  |
| --- | --- | --- | --- | --- | --- | --- | --- | --- | --- | --- |
| Scholarly activities | 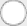 |  | 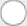 |  | 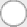 |  | 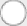 |  | 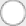 |  |
|  |  |  |  |  |  |  |  |  |  |  |

1. Please rate your satisfaction with how your current job has opened other academic career opportunities for you.

3- Neither

2- Moderately dissatisfied/nor 4- Moderately

1- Very dissatisfied dissatisfied satisfied satisfied 5- Very satisfied N/A


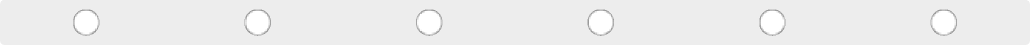


5

Job Diagnostic Survey for Clinician-Educators

**Section III: Please provide following additional information about yourself.**

1. Gender


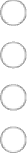


Male

Female

Other

Do not want to disclose

1. What is your age?


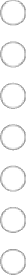


25 to 34

35 to 44

45 to 55

55 to 64

65 to 74

75 or older

Do not want to disclose

1. How long (number of years) have you served as a CE?

6

Job Diagnostic Survey for Clinician-Educators

**Section IV: The following question pertain to the characteristics of your institution.**

1. Approximately, how many trainees are there in your department?
2. Approximately, how many CE's are there in your department?

7
