# Supplementary material for: Job characteristics that enrich clinician-educators’ career: a theory-informed exploratory survey
Source: Med Educ Online. 2022 Dec 22;28(1):2158528. doi: 10.1080/10872981.2022.2158528 (PMC9793935; doi:10.1080/10872981.2022.2158528)
Supplement: Supplemental Material [file ZMEO_A_2158528_SM2569.zip › Supplementary files/Supplemental 2.docx]

**Supplemental - 2**

| Characteristic | Motivating Potential Score | | Total Job Satisfaction Score | |
| --- | --- | --- | --- | --- |
|  | Mean (SD) | P-value^a^ | Mean (SD) | P-value^a^ |
| Advanced Degree(s)  No  Pursuing  Yes | 63 (25)  53 (20)  59 (21) | 0.40 | 18 (3)  16 (3)  18 (4) | 0.12 |
| Age  25 to 34  35 to 44  45 to 55  55 to 64  65 to 74 | 60 (17)  53 (23)  62 (25)  69 (24)  62 (22) | 0.008 | 18 (3)  17 (3)  18 (3)  19 (3)  18 (4) | 0.08 |
| Gender  Female  Male | 63 (25)  60 (23) | 0.34 | 18 (4)  18 (3) | 0.74 |
| Education Leadership Role  Yes  No | 65 (26)  60 (23) | 0.20 | 18 (3)  18 (4) | 0.21 |
| Other Leadership Role  Yes  No | 60 (23)  18 (3) | 0.43 | 63 (22)  18 (3) | 0.06 |
| Protected time for educational service(s)  Yes  No | 61 (24)  18 (4) | 0.75 | 62 (23)  18 (3) | 0.71 |
|  | Pearson correlation coefficient | P^b^ | Pearson correlation coefficient | P^b^ |
| Number of years of experience as Clinician Educator | 0.15 | 0.04 | 0.18 | 0.01 |

Supplemental 2. Bivariate Associations: Motivating Potential Scores and Total Job Satisfaction Scores by Clinician Educators Demographics (n=201)

^a^P-value represents independent samples t-test or analysis of variance for trend.

^b^P-value denoting significance of Pearson correlation coefficient.
