# Supplementary material for: Job characteristics that enrich clinician-educators’ career: a theory-informed exploratory survey
Source: Med Educ Online. 2022 Dec 22;28(1):2158528. doi: 10.1080/10872981.2022.2158528 (PMC9793935; doi:10.1080/10872981.2022.2158528)
Supplement: Supplemental Material [file ZMEO_A_2158528_SM2569.zip › Supplementary files/Supplemental 3.docx]

**Supplemental - 3**

| Simple linear regression | Coefficient  (95% CI)^a^ | R-square | P-value^b^ |
| --- | --- | --- | --- |
| Advanced Degree(s)  (ref = no), n=196 | -0.87 (-1.89, 0.15) | 0.014 | 0.10 |
| Age, n=194 | 0.34 (-0.09, 0.77) | 0.012 | 0.12 |
| Gender (ref = male), n=194 | -0.16 (-1.12, 0.80) | 0.000 | 0.74 |
| Leadership Role(s)  (ref = no), n=196 | 0.69 (-0.38, 1.76) | 0.008 | 0.20 |
| Other Leadership Role(s) (ref = no) | 0.91 (-0.04, 1.86) | 0.018 | 0.06 |
| Protected time for educational service(s)  (ref=no), n=196 | 0.20 (-0.86, 1.26) | 0.000 | 0.71 |
| Number of years as a clinician-educator, n=196 | 0.06 (0.01, 0.11) | 0.032 | 0.01 |
| Skill variety, n=196 | 1.31 (0.71, 1.91) | 0.086 | <.0001 |
| Task Identity-Well Defined, n=196 | 0.64 (0.15, 1.13) | 0.033 | 0.01 |
| Task Identity-Visible/Identifiable, n=196 | 0.92 (0.40, 1.44) | 0.058 | 0.0006 |
| Task Significance | 0.52 (-0.09, 1.14) | 0.014 | 0.09 |
| Autonomy | 1.1 (0.50, 1.73) | 0.062 | 0.0005 |
| Feedback-Internal | 1.40 (0.92, 1.90) | 0.144 | <.0001 |
| Feedback-External | 0.79 (0.31, 1.28) | 0.051 | 0.0014 |
| Motivating Potential Score | 0.06 (0.04, 0.08) | 0.16 | <.0001 |
| Multiple Linear Regression | Unstandardized coefficient | Standardized β | P-value^b^ |
| Final Model 1^c^  Intercept  Skill variety  Task Identity-Well Defined  Autonomy  Feedback-Internal  Number of years as a clinician-educator | 5.90  0.67  0.44  0.52  1.20  0.05 | 0.15  0.13  0.12  0.33  0.14 | 0.02  0.05  0.08  <.0001  0.03 |
| Final Model 2^d^  Intercept  MPS  Number of years as a clinician-educator  Other formal Leader (Ref=yes) | 13.79  0.05  0.04  0.77 | 0.39  0.11  0.12 | <.0001  0.10  0.08 |

Supplemental 3: Simple and Multiple Linear Regression Models for the Total Job Satisfaction Score of Clinician Educators. To predict total job satisfaction score, descriptive predictors, and individuals items for the Motivating Potential in Model 2.

^a^ Unstandardized beta coefficient, with 95% Confidence Interval (CI)^, b^P-value indicates coefficient significance

^c^R-square =0.275

^d^R-square = 0.198
